# IMPACT OF INFORMAL AND FORMAL SOCIAL NETWORKS DURING A WILDFIRE ON PSYCHOLOGICAL RECOVERY

**DOI:** 10.1093/geroni/igz038.1435

**Published:** 2019-11-08

**Authors:** Judith Robertson R Phillips

**Affiliations:** California State University San Marcos, San Marcos, California, United States

## Abstract

Having the support of a social network can play an important role towards psychological well-being for those impacted by a disaster. The purpose of this study was to investigate how type (emotional, tangible, informational) and source (family, friends, others) of social support during three Southern California wildfires impacted the psychological well-being of three victim-groups (based on severity of exposure) of community-residing adults (N= 203; meanage = 63.4 years; range 50-94 years) who responded to surveys about their experiences during the 2007, 2014, and 2017 northern San Diego County, CA wildfires. Analyses revealed that emotional social support from friends and neighbors was the most frequently received type and source of support, especially for the primary victim-group, those with residential loss. All victim-groups exhibited healthy psychological well-being. Discussion will focus on how informal groups differed and were similar to formal groups in providing social support during a disaster to encourage psychological recovery.

